# Investigation of altered retinal microvasculature in female patients with rheumatoid arthritis: optical coherence tomography angiography detection

**DOI:** 10.1042/BSR20230045

**Published:** 2023-10-13

**Authors:** Hsuan-Yi Lee, Jun Chen, Pin Ying, San-Hua Xu, Min Kang, Jie Zou, Xu-Lin Liao, Wenqing Shi, Qian Ling, Yi-Xin Wang, Hong Wei, Yi Shao

**Affiliations:** 1 Department of Ophthalmology, The First Affiliated Hospital of Nanchang University, Jiangxi Branch of National Clinical Research Center for Ocular Disease, Nanchang, Jiangxi 330006, China; 2 Department of Optometric Medicine and Ophthalmology, The Nanchang University, Nanchang, Jiangxi 330006, P.R. China; 3 Department of Ophthalmology and Visual Sciences, The Chinese University of Hong Kong, Shatin, New Territories, Hong Kong 999077, China; 4 School of Optometry and Vision Science, Cardiff University, Cardiff, CF24 4HQ, Wales

**Keywords:** Optical coherence tomography angiography (OCTA), Rheumatoid arthritis (RA), vascular density

## Abstract

Background: Rheumatoid arthritis (RA) is a chronic, systemic autoimmune disorder that primarily causes symmetrical polyarthritis and bone deformity. In RA patients, sight-threatening inflammatory eye complications would be expected.

Objective: The objective of the study is to ascertain the macular retinal vessel density changes in RA patients and controls using optical coherence tomography angiography (OCTA), and to investigate the association between disease and microvascular density alterations.

Methods: A total of 12 RA patients (24 eyes) and 12 age- and gender-matched control participants (24 eyes) were recruited to the study. We used the Early Treatment Diabetic Retinopathy Study partitioning, hemispheric quadrants and annular partitioning to segment each image into different subregions. The vascular density of superficial retina layer, deep retina layer and conjunctival capillary plexus was quantitatively measured by OCTA and compared with the control group. Correlation analysis was used to explore the relationship between STMI and conjunctival capillaries densities.

Results: In the superficial retinal layer, the vascular density of S, I, L, SL, SR, IL and C1-C5 were significantly decreased in the RA group compared with the control group (*P*<0.05). For the deep retinal layer, the vascular density of S, SL, SR, IL, C1, C2 and C4 also decreased in RA group. A significant positive correlation was indicated between conjunctival vascular and STMI densities (*r* = 0.713, *P*<0.05).

Conclusion: OCTA results suggest that RA patients present with a reduced macular retinal vascular density. These subtle alterations of ocular microcirculation may precede severe eye involvements and may be a potential biomarker for early distinguishing abnormal eyes from healthy eyes.

## Introduction

Rheumatoid arthritis (RA) is a multisystem autoimmune disorder that predominately presents with a symmetrical polyarthritis of synovial joints but includes extra-articular manifestations and complications due to the chronic, complicated, inflammatory and autoimmune characteristics of RA [1–3]. According to the report, the prevalence is approximately from 5 to 50 per 100,000 people, the highest incidence rate is among women of age approximately 50 years old [4]. Epidemiologic statistics suggested RA and gender to be substantially related, with an age-dependent confirmed 3-fold increased disease incidence in females compared with males. Multifactor synergism, hormonal variables and genetic expressions, may account for this sexual discrepancy in RA prevalence [5]. Most notably, several prospective cohort studies revealed that females with RA had generally higher baseline disease activity scores (DAS) and a 3-fold faster disease exacerbation than males [32,33]. Although synovitis is the typical pathological hallmark of RA, the chronic inflammatory state can induce many extra-articular involvements and comorbidities such as rheumatoid nodules and vasculitis as well as to ocular, pulmonary, neurological, cardiovascular and hematologic disease [6–12]. There is much evidence that RA is known to be related to accelerated atherosclerosis and excessive cardiovascular risk due to chronic inflammation and autoimmune status [13–15]. According to the research [16], the atherogenesis induced by RA is pertinent to systemic microvascular dysfunction that subsequently impacts myocardial perfusion and causes myocardial ischemia, not only present with the formation of atherosclerotic plaque in large conduit arteries. However, patients suffering from RA rarely show typical clinical signs and symptoms, even among patients without cardiovascular complications also exhibit impaired myocardial perfusion [17,18]. In fact, the ocular involvement is a common symptom of RA and is even associated with potentially sight-threatening inflammatory eye disease. Sometimes, it may be the premonitory symptom of the disease. The main spectrum of ocular abnormality comprises episcleritis, scleritis, choroidal thinning and keratoconjunctivitis sicca [19–22]. Among these, dry eye is the most frequent ophthalmological complication, affecting approximately one-quarter of RA patients. The secondary Sjögren’s syndrome due to long-term dry eye can cause the lesion to the structure of conjunctival epithelial cells [23,24]. Indeed, the changes of the large arteries and peripheral microvascular circulation in RA are substantiated with implications of autoantibody-mediated inflammation and cell-mediated inflammation. The unique structure of retinal vasculature that exists as a potential similarity to cardiovascular microcirculation in terms of physiological and anatomical features, enables rapid and non-invasive visualization of the subtle alterations of microcirculation in humans in vivo. Therefore, the macular retinal microcirculation secondary to autoimmune rheumatic disorders can serve as a sign of pathological vascular abnormality and provide a hint for early systemic pathophysiological alterations induced by disease. Taking advantage of these features, quantification of structural alteration in the macular retinal microvasculature may represent a novel biomarker for generalized microvascular dysfunction caused by RA. We hypothesized that there is a morphologic alteration in macular retinal microvasculature that played a role in observing the evolution of the inflammatory and autoimmune rheumatic disease and it is therefore of primary importance to identify the microvascular density of the retinal microvascular stratification before proving that RA causes the lesion in macular retinal microvasculature.

Fluorescein angiography (FA) is the main detection method to retinal ischemia and microvascular changes in retinopathy over the years, but its invasive diagnosis and subsequent adverse effect limit its clinical application. The optical coherence tomography angiography (OCTA) is a noninvasive, clinically feasible high-resolution technique for examination of the fine retinal structure of the retina. This imaging technique allows for quantitative analysis of the retinal vasculature and provides a method for measuring the prominence or excavation of the optic disc. Meanwhile, OCTA has evolved into a feasible imaging modality for understanding a variety of retinopathy, including age-related macular degeneration and choroidal neovascular membranes. OCTA has also been used for the diagnosis of ocular complications such as systemic lupus erythematosus, arteritic and non-arteritic optic neuropathy, Alzheimer’s disease, abnormalities in multiple sclerosis, thyroid-related eye disease, and Parkinson's disease [25–28].

## Methods

### Research subjects

The 12 patients initially diagnosed with RA (24 eyes) as defined by the 2010 American College of Rheumatology criteria or European League Against Rheumatism criteria (ACR-EULAR) and 12 age- and gender-matched normal controls (24 eyes) were recruited in this cross-sectional study from the First Affiliated Hospital of Nanchang University. The control group was composed of healthy participants with no autoimmune disorder or ophthalmic damage which could cause the abnormalities of eye circulation. All participants voluntarily underwent an OCTA and hematology examination to identify the presence of ocular of systemic disease before enrollment into the study. The Ophthalmological examination and OCTA were conducted in all participants by the same ophthalmologist.

RA patients satisfying the following criteria were recruited in present study: (1) first diagnosed with rheumatoid arthritis based on the 2010 ACR-EULAR criteria with over 5 years disease duration, (2) female, (3) naked eye or best corrected visual acuity >1.0, (4) admit to no history of taking any glucocorticoids and chloroquine before the receiving examination. Participants satisfying any of the following were excluded from the present study: (1) diagnosed with other autoimmune disorders, (2) other inflammatory joint diseases, such as reactive arthritis, (3) other chronic disease, such as diabetes and hypertension, (4) retinopathy diseases other than RA, such as age-related macular degeneration or eye vascular occlusion, (6) active ocular inflammation, such as uveitis and optic neuritis, (5) systemic vascular diseases, (6) ophthalmic surgery within half year, (7) pregnancy or lactation participants, and (8) contraindication, hypersensitivity or intolerance to local anesthetics or mydriatics.

### Ethical considerations

The research methods of this study were confirmed to the principles of the 1964 Declaration of Helsinki and approval by the ethics committee of the First Affiliated Hospital of Nanchang University. All the participants signed an informed consent form after realizing the methods, purpose and potential risk of the present study.

### Clinical examination

All participants underwent ophthalmological evaluations including habitual visual acuity testing, intraocular pressure measurement, fundus photography, and OCTA scan (Cirrus 5000, version 10.0; Zeiss Meditec, California, U.S.A.). For OCTA scan, the 6 × 6 mm scan pattern was conducted at the macular retina to real-time quantitative evaluation retinal vascular circulation. Erythrocyte sedimentation rate (ESR), C-reactive protein (CRP), disease activity index (SLEDAI-2K), autoantibody testing and disease duration were recorded at baseline. The demographic characteristics and clinical indicators were summarized in Table 1.

**Table 1:** Clinical and demographic characteristics of participants with RA and controls

Parameters	Group		*t*	*P*
	HC (*n*=12)	RA (*n*=12)		
Age (year)				
Mean ± SD	38.82 ± 5.73	39.00 ± 9.85	0.022	0.98^†^
Range	(28–47)	(28–49)	–	–
Gender (male/female)	0/ 12	0/12	N/A	1.00^‡^
Average Visual acuity	0.89 ± 0.12	0.68 ± 0.23	2.315	0.03^*^†
Average IOP (mmHg)	14.89 ± 1.06	15.36 ± 1.57	0.928	0.36 ^†^
ESR (mm)	3.84 ± 1.24	15.93 ± 8.43	4.969	<0.001^*†^
CRP (10 mg/L)	1.31 ±0.77	3.27 ± 2.44	1.648	<0.01^*†^
ANA+	N/A	15/15	–	–
APA+	N/A	0/15	–	–
SLEDAI-2K	N/A	3.68 ± 2.32	–	–
SDI	N/A	0.26 ± 0.46	–	–
Blood pressure				
SBP (mmHg)	126.92 ± 16.75	125.08 ± 6.14	0.73	0.48^†^
DBP (mmHg)	81.57 ± 6.80	80.6 ± 8.35	0.351	0.73^†^
Disease duration (years)	N/A	8.84 ± 3.12	–	–

* *P*<0.05; ^†^, independent sample *t*-test; ^‡^, Pearson chi-square test.

ANA, antinuclear antibody; APA, antiphospholipid antibody; CRP, C-reactive protein; DBP, diastolic blood pressure; ESR, erythrocyte sedimentation rate; HC, healthy control; IOP, intraocular pressure; RA, rheumatoid arthritis; SBP, systolic blood pressure; SD, standard deviation; SDI, Systemic Lupus Erythematosus International Cooperative Clinic/American College of Rheumatology Injury Index; SLEDAI-2K, Systemic Lupus Erythematosus Disease Activity Index -2000.

### OCTA

All OCTA measurements were conducted by the same ophthalmologist using the OCTA imaging with RTVue Avanti XR system (Optovue, Fremont, CA, U.S.A.). The OCTA used in the present study operates at 70,000 a scans per second, along with a superluminescent diode light source of an 840 nm central wavelength. An axial resolution of 5 μm and the lateral resolution was approximately 22 μm. Within 3.9 s. Over an approximated 6 × 6 mm retinal region centered on the fovea, two volumes scans were taken sequentially using orthogonal raster scan patterns (denoted, respectively, *x*-fast and *y*-fast). Each B-scan for the *x*-fast scan pattern consisted of 216 A-scans (along the *x*-axis), and two successive B-scans were taken at each of the 216 places along the *y*-axis of the cube. We acquired 1080 B-scans (216 *y*-positions×5 B-scans) at the speed rate of 270 frames per second. The OCTA scans were automatically aligned and merged by Motion Correction Technology (MCT) to eliminate motion artifacts and produced the OCTA images. Finally, we obtained a three-dimensional 6×6 mm en-face OCTA angiographic image for each eye [29]. All participants received OCTA scans during the same time to avoid diurnal variations that may affect the results of ocular examination.

**Figure 1:**
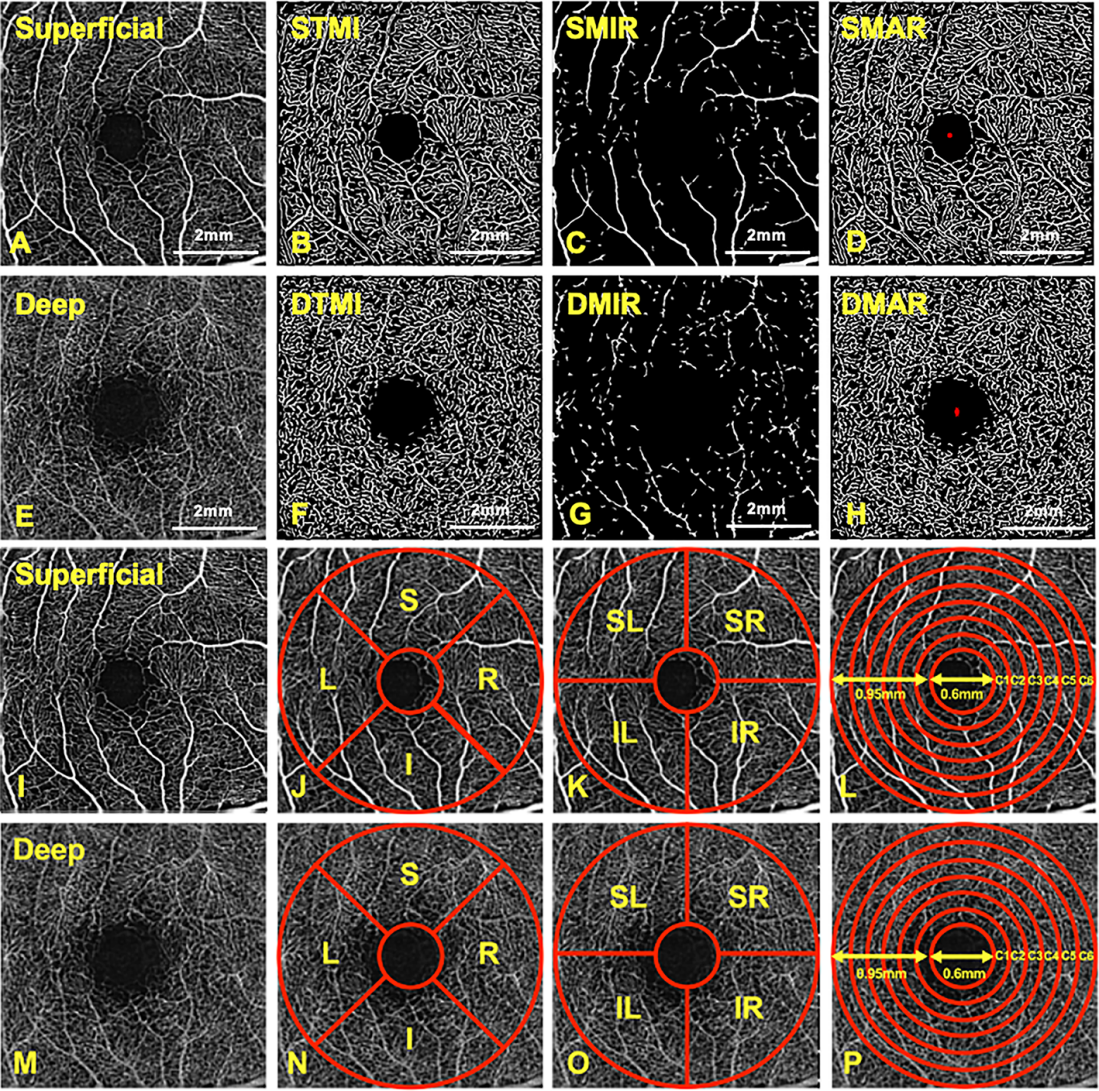
The retinal 6× 6mm OCTA images at the macular area and different partition methods (**A,I**) Superficial retinal microvessel density image. (**B**) STMI: superficial total microvascular. (**C**) SMIR: superficial microvascular. (**D**) SMAR: superficial macrovascular. (**E,M**) Deep retinal microvessel density image. (**F**) DTMI: deep total microvascular. (**G**) DMIR: deep microvascular. (**H**) DMAR: deep macrovascular. (**J–L**) Three different partition methods in the superficial retinal layers. (**N–P**) Three different partition methods in the deep retinal layers.

We artificially segment the macular retinal capillary bed into two distinct physiologic layers based on the slit-spectrum amplitude-decorrelation algorithm: (1) A superficial retinal layer (SRL, the layer between the vitreous retinal in interface and the anterior boundary of the ganglion cell layer) (Figure 1A–D,I–L) (2) A deep retinal layer (DRL, the layer between the outer boundary of the outer plexiform layer and the inner boundary of the inner plexiform layer) (Figure 1E–H,M–P). Also, we analyzed the densities of superficial microvascular (SMIR; Figure 1C) and deep microvascular (DMIR; Figure 1G), **superficial** macrovascular (SMAR; Figure 1D) and deep macrovascular (DMAR; Figure 1H) and superficial total microvascular (STMI; Figure 1B) and deep total microvascular (DTMI, Figure 1F) networks in the two stratifications. After scanning, we segmented each OCTA image as follows [29] (Figure 1I–P): (1) Early Treatment of Diabetic Retinopathy Study (ETDRS) subdivision regions, consisting of S, I, R, and L quadrants in superficial (Figure 1J) and deep (Figure 1N) layers. (2) Hemispheric segmentation method, image division based on horizontal and vertical diagonals, including SL, SR, IL, and IR subregions in superficial (Figure 1K) and deep (Figure 1O) layers. (3) Central annuli segmentation method. After removing the avascular area of the central fovea with a diameter of 0.6 mm, the circular area with a diameter of 2.5 mm on the outside was divided into a circular area with a bandwidth of 0.95 mm (C1–C6). The circular area is segmented into six narrow rings with a 0.16 mm bandwidth in superficial (Figure 1L) and deep (Figure 1P) layers.

### Fractal dimension calculation

In this cross-sectional study, we propose an ImageJ (National Institutes of Health, Bethesda, MD, U.S.A.) software that allows researchers to measure microvascular parameters from the OCTA images. First, the background noise was muted using a nonlocal means denoising filter. Second, the box-counting method provided by the plugin of ImageJ program was used to analyze gray-scale images for the fractal dimension [30]. Box-counting method depends on the formula $N\propto \varepsilon^{Df}$, among *N* is the number of targets, ɛ is the amplification factor or linear scaling, and *Df* is the fractal dimension. A log- log plot of ɛ and *N* can be used to assess the fractal dimension (*Df*) as shown below: *Df* = log*N*/log*ɛ* [31].

### Statistical analysis

All data were statistically analyzed with SPSS version 22.0 (IBM, Armonk, NY, U.S.A.) and GraphPad Prism version 9.0 (GraphPad Software, La Jolla, CA, U.S.A.), and presented as mean ± SD. Independent *t*-test, Pearson chi-square test and Fisher’s exact test were conducted to compare the demographic characteristics between RA and control groups. The discrepancies of microvessel densities in the retinal subregions were analyzed by one-way analysis of variance (ANOVA). Linear correlation analysis was used to analyze the relationship between conjunctival capillary plexus and STMI density. The microvessel densities of the superficial and deep retinal layers between groups were compared with the receiver operating characteristic (ROC) curve to distinguish the RA patients and healthy controls. *P*<0.05 was deemed statistically significant.

## Results

### Demographic characteristics of participants

The baseline characteristics of participants were recorded to identify the similar aspects in the two groups, as shown in Table 1. There were 12 participants (24 eyes) in each group. The mean age (the RA group was 39.00 ± 9.85 and the HC group was 38.83 ± 5.73 years; *P*=0.98) and gender ratio (12 females in each group; *P*>0.99) were statistically similar in two groups. In the RA group, the ESR was 15.93 ± 8.43 (*P*<0.001) and CRP was 3.27 ± 2.44 (*P*<0.01). While the visual acuity of RA patients was statistically worse than that of the HCs (*P*=0.03).

### Analysis of superficial retinal microvessel density

**Figure 2:**
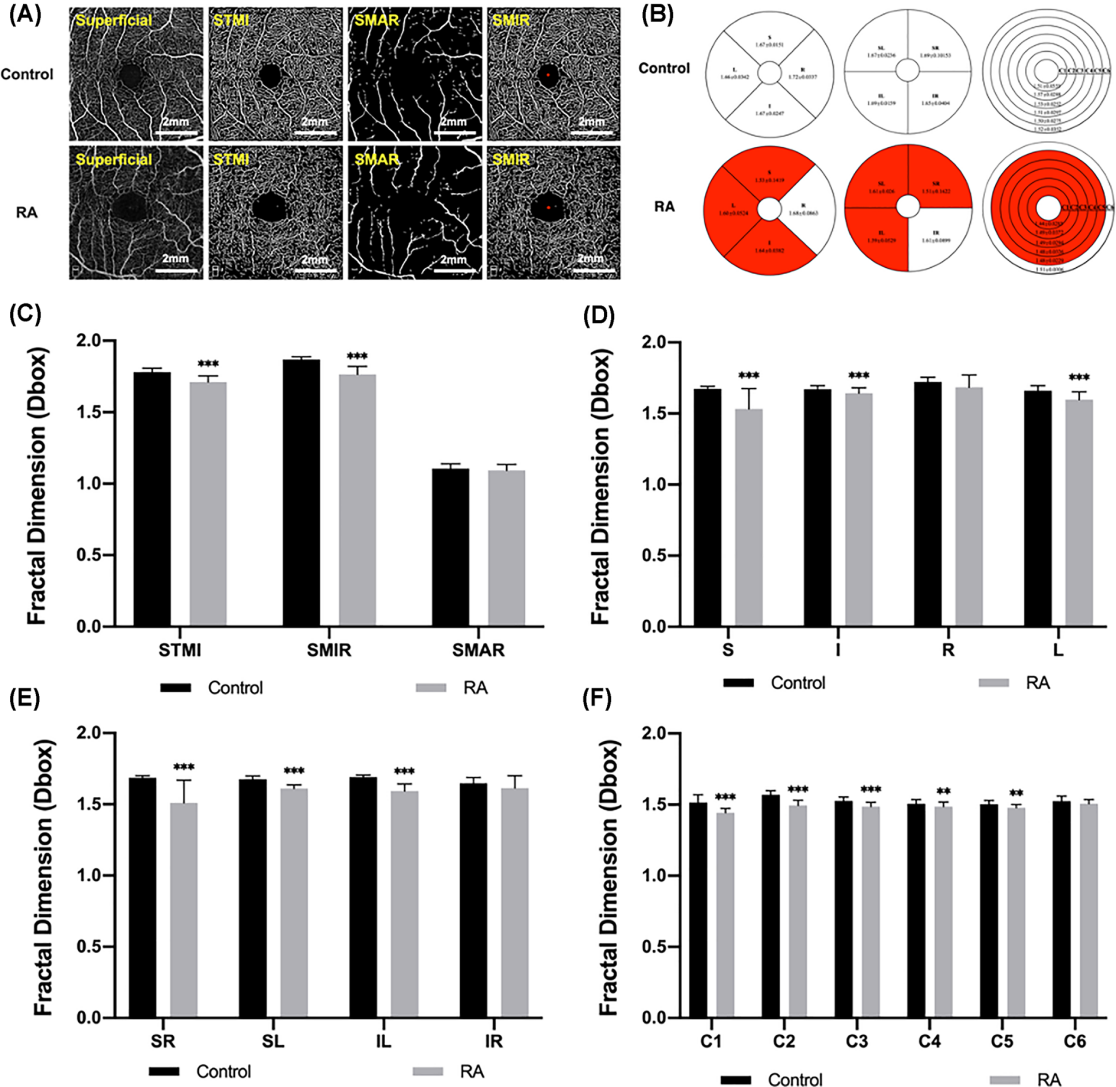
The macular retinal microvessel density analysis of superficial retinal layer in RA and control groups (**A**) The OCTA images of superficial retinal vessel density. (**B**) Comparison of retinal vessel density in three partition methods between two groups. (**C–F**) Results of superficial retinal vessel density analysis in each subregion between groups. **P<0.01; ***P<0.001.

A comparison of the subregional superficial retinal vessel density was conducted among groups, the measurements were shown in Table 2. Comparing the MIR, MAR and TMI densities in the superficial retinal layer, we observed a significant decrease in MIR and TMI of the RA group compared with the HC group (*P*<0.001), but no significant change in MAR density (*P*=0.253; Figure 2A,C). Using the ETDRS partitioning, it was found that the microvessel density at the S, I (*P*=0.005) and L subregions in RA group was significantly lower than that in HC group (*P*<0.001; Figure 2B,D). In the hemispheric segmentation method, we observed that the SL, SR and IL subregional densities were statistically decreased in the patients with RA (*P*<0.001; Figure 2B,E). Similarly, using the central annuli segmentation method, the microvessel density in the C1-C3 (*P*<0.001), C4 (*P* =0.032), and C5 (*P* =0.018) regions was significantly lower than that in the HC group (Figure 2B,F). In the areas not highlighted here the densities of two groups were no statistically different.

**Table 2:** Comparison of superficial retinal microvessel density at different subregions among the groups

Subregion	HC group (*n*=12; 28 eyes)	RA group (*n*=12; 28 eyes)	*P*
MIR	1.88 ± 0.017	1.72 ± 0.07	**<0.001**
MAR	1.11 ± 0.033	1.09 ± 0.026	0.253
TMI	1.78 ± 0.027	1.71 ± 0.052	**<0.001**
Early Treatment Diabetic Retinopathy Study (ETDRS)			
S	1.67 ± 0.015	1.53 ± 0.142	**<0.001**
I	1.67 ± 0.025	1.64 ± 0.038	0.**005**
L	1.66 ± 0.034	1.60 ± 0.052	**<0.001**
R	1.72 ± 0.034	1.68 ± 0.086	0.231
Hemispheric segmentation method			
SL	1.67 ± 0.024	1.61 ± 0.026	**<0.001**
SR	1.69 ± 0.102	1.51 ± 0.162	**<0.001**
IL	1.69 ± 0.016	1.59 ± 0.053	**<0.001**
IR	1.65 ± 0.040	1.61 ± 0.090	0.093
Central annuli segmentation method			
C1	1.51 ± 0.056	1.44 ± 0.029	**<0.001**
C2	1.57 ± 0.029	1.49 ± 0.037	**<0.001**
C3	1.53 ± 0.025	1.49 ± 0.029	**<0.001**
C4	1.51 ± 0.029	1.48 ± 0.033	0.**032**
C5	1.50 ± 0.027	1.48 ± 0.028	0.**018**
C6	1.52 ± 0.035	1.51 ± 0.031	0.305

### Analysis of deep retinal microvessel density

**Figure 3:**
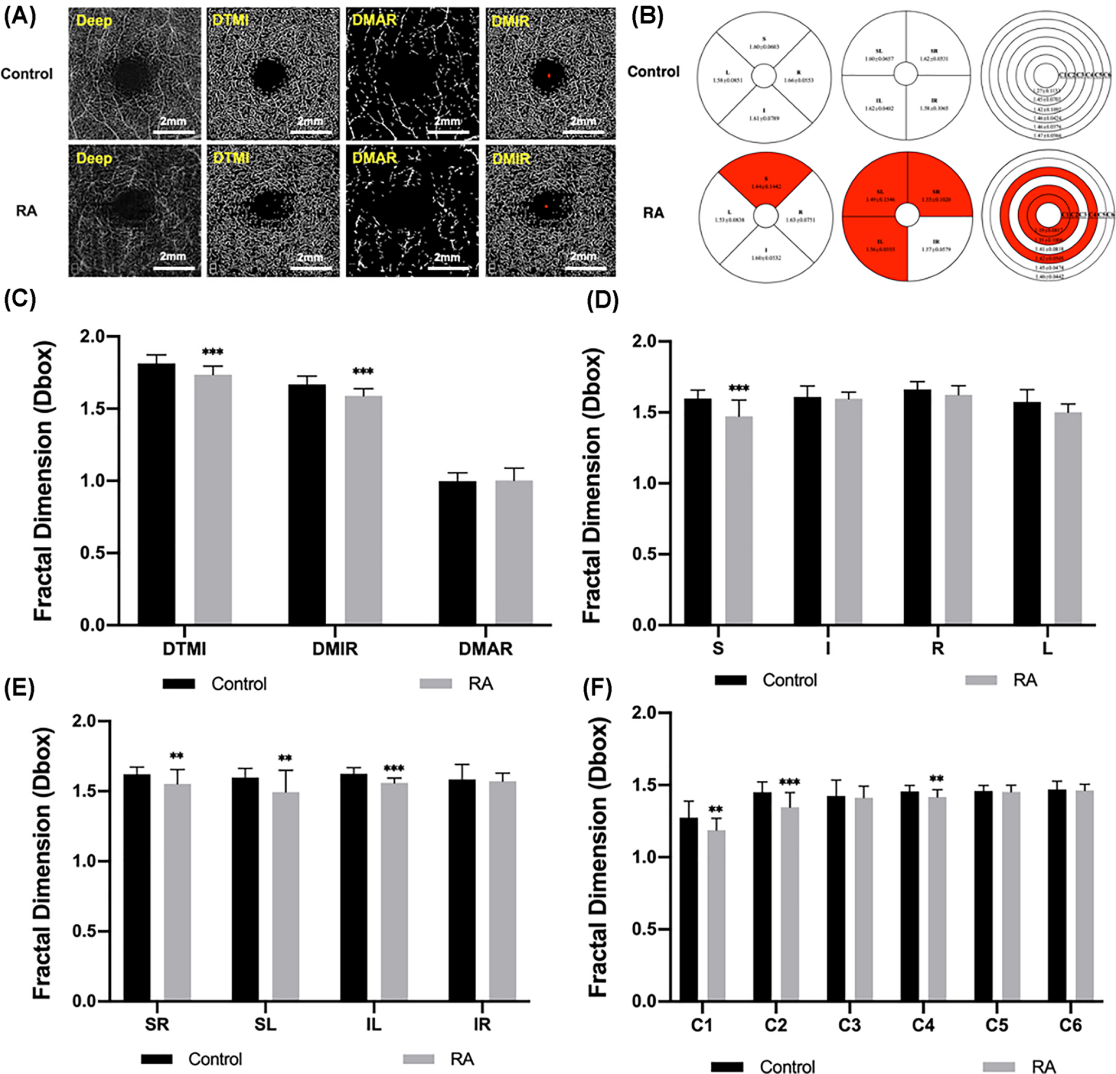
The macular retinal microvessel density analysis of deep retinal layer in RA and control groups (**A**) The OCTA images of deep retinal vessel density. (**B**) Comparison of retinal vessel density in three partition methods between two groups. (**C–F**) Results of deep retinal vessel density analysis in each subregion between groups. **P<0.01; ***P<0.001.

Comparisons of measurements of deep retinal vessel density were shown in Table 3. The densities of MIR, MAR and TMI in deep retinal layer were compared between RA and HC groups. Similarly, we observed a significant decrease in MIR and TMI of the RA group compared with the HC group (*P*<0.001), but no significant change in MAR density (*P*=0.839; Figure 3A,C). Using the ETDRS partitioning, it was found that the microvessel density at the S subregion in RA group was significantly lower than that in HC group (*P*<0.001; Figure 3B,D). In the hemispheric segmentation method, we observed that the SL, SR (*P*=0.007) and IL subregional densities were statistically decreased in the patients with RA (*P*<0.001; Figure 3B,E). Using the central annuli segmentation method, the microvessel density in the C1 (*P*=0.006), C2 (*P*<0.001) and C4 (*P*=0.006) regions was significantly lower than that in the HC group (Figure 3B,F). Similarly, in the areas not highlighted here the densities of the two groups were no statistically different.

**Table 3:** Comparison of deep retinal microvessel density at different subregions among the groups

Subregion		HC group (*n*=12; 28 eyes)	RA group (*n*=12; 28 eyes)	*P*
MIR		1.67 ± 0.056	1.57 ± 0.039	**<.001**
MAR		1.00 ± 0.057	1.00 ± 0.078	.839
TMI		1.81 ± 0.053	1.73 ± 0.054	**<.001**
Early Treatment Diabetic Retinopathy Study (ETDRS)				
S		1.60 ± 0.060	1.44 ± 0.144	**<.001**
I		1.61 ± 0.079	1.60 ± 0.053	.132
L		1.58 ± 0.085	1.53 ± 0.084	.087
R		1.66 ± 0.055	1.63 ± 0.075	.261
Hemispheric segmentation method				
SL		1.60 ± 0.066	1.49 ± 0.155	**<.001**
SR		1.62 ± 0.053	1.55 ± 0.102	.**007**
IL		1.62 ± 0.040	1.56 ± 0.036	**<.001**
IR		1.58 ± 0.107	1.57 ± 0.058	.153
Central annuli segmentation method				
C1		1.27 ± 0.113	1.19 ± 0.082	.**006**
C2		1.45 ± 0.070	1.35 ± 0.101	**<.001**
C3		1.42 ± 0.109	1.41 ± 0.082	.095
C4		1.46 ± 0.042	1.42 ± 0.051	.**006**
C5		1.46 ± 0.038	1.45 ± 0.047	.114
C6		1.47 ± 0.057	1.46 ± 0.044	.422

### ROC analysis of superficial and deep retinal microvessel densities

**Figure 4:**
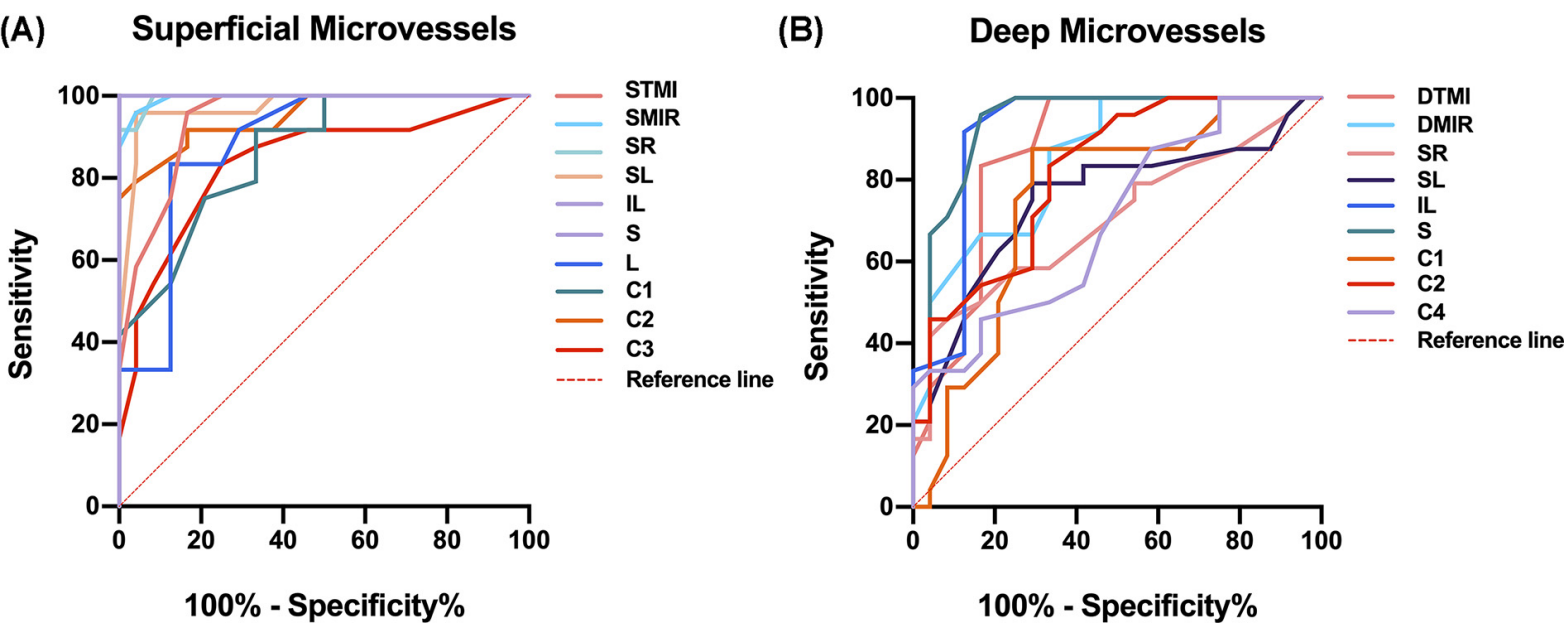
ROC curve analysis of different retinal subregional microvascular densities in two stratifications (**A**) The ROC curve analysis for superficial retina. (**B**) The ROC curve analysis for deep retina.

The macular retinal microvessel density as measured by OCTA indicated the sensitivity and specificity to distinguish the RA from normal controls. In the superficial retina, the MIR, TMI, S, I, L SL, SR, IL, and C1-C6 subregions showed a statistical difference between the two groups. Among them, the areas under the curve (AUC) of the S and IL regions of the retinal vessel density were 1.00 [95% confidence interval (CI) = 1.00), showing that it had high diagnostic sensitivity for the retinal microvascular lesion caused by RA (Figure 4A). Compared with the HC group, there was a significant difference in the MIR, TMI, S, SL, SR, IL, C1, C2, and C4 regions in the deep retinal layer in the RA group. The AUC of deep retinal vessel density of the S region were the largest, which was 0.93 [95% confidence interval (CI) = 0.87–1.00], indicating that the diagnosis of RA by superficial S region had high sensitivity (Figure 4B).

### Relationship between STMI density and conjunctival capillary density

**Figure 5:**
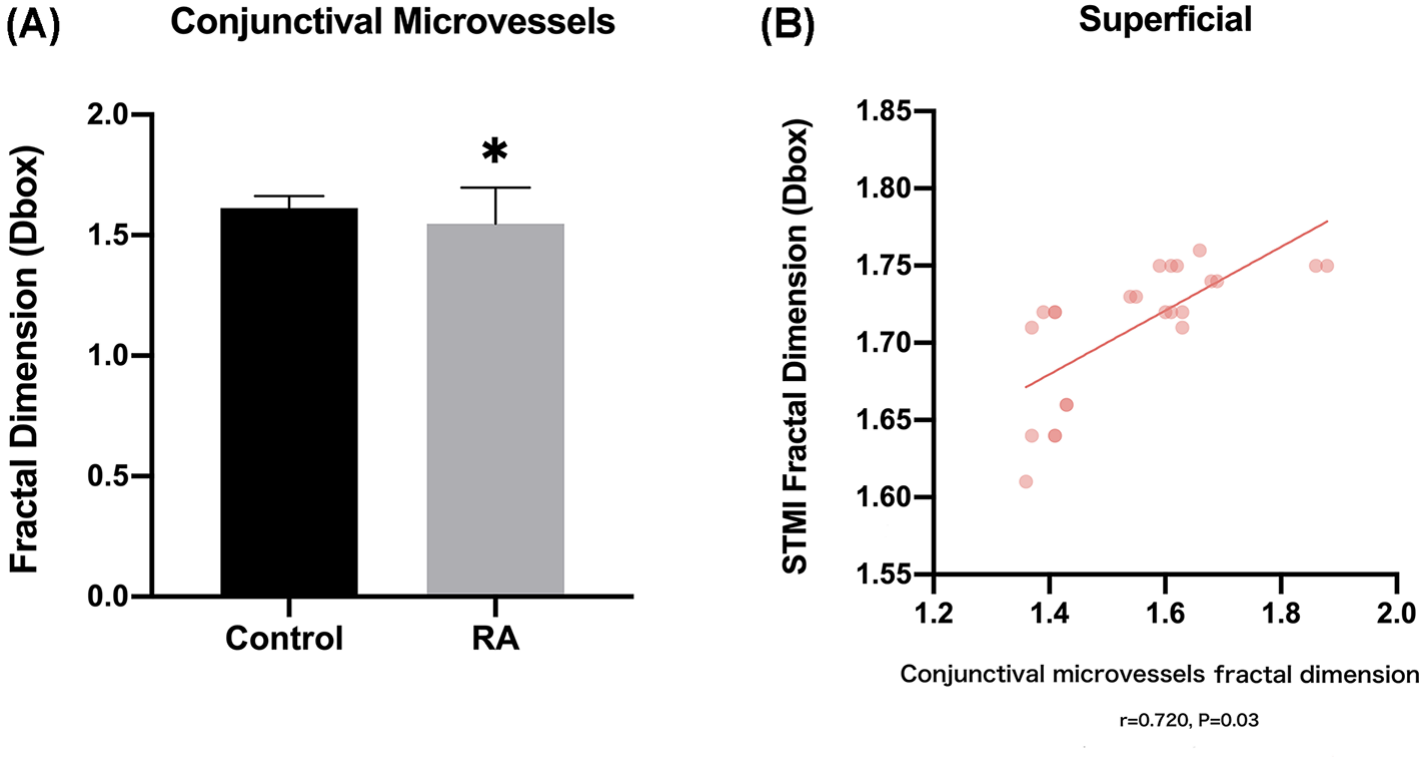
The analysis of temporal conjunctival density between RA and control groups (**A**) Histogram analysis of conjunctival capillary density in the groups. (**B**) The correlation analysis between superficial total microvascular (STMI) and conjunctival capillary in RA and control groups. *P<0.05.

In the RA group, the conjunctival capillary density was significantly lower than the HC group (*t*=2.040; *P*=0.033; Figure 5A). Evaluating the relationship between superficial retinal and conjunctival capillary densities, we observed that the correlation coefficient of TMI network and temporal conjunctival density was 0.720 (Figure 5B).

## Discussion

The present study provides to a pinpoint analysis of the relationship between RA and microcirculation of macular retinal vessel changes. The retinal microvascular abnormalities presented in OCTA images may facilitate early detection for potential eye involvements due to rheumatic disease and may serve as a biomarker for distinguishing abnormal retinal microcirculation from those healthy retinas. In this cross-sectional study, the densities of S, I, L, SL, SR, IL, C1-C5 quadrants in the superficial retina layer of RA patients was statistically lower than that in HC group (*P*<0.05), and the densities of S, SL, SR, IL, C1-C2, and C4 quadrants in the deep capillaries was statistically lower than HC group (*P*<0.05). To our knowledge, few studies have been conducted in the issue of retinal microvascular alterations and female RA. However, the prospective cohort study with 332 early RA patients revealed that the DAS score and rate of disease progression were all statistically higher in women than men, despite the same radiographic progression at three years of follow-up [32]. Similarly, the higher DAS score and health assessment questionnaire disability index (HAQ-DI) in females compared with males were also reported in the cross-sectional analysis of over 6000 RA patients from 25 countries (DAS: 4.3 vs. 3.8; HAQ-DI: 1.1 vs. 0.8) [33]. Therefore, identifying these alterations in retinal microcirculation prior to the disease progression to disability by OCTA may be a feasible method for facilitating the early distinguishing of abnormal eye affected by RA from those normal eyes and improving the prognosis of the female patients with ocular involvements. Our study is one of the few studies investigating the patterns of retinal microvascular changes in the female with RA by OCTA.

Retinal vasculitis is the pathological basis of retinopathy induced by RA [34]. The excessive inflammatory substance produced by cell, autoantibody and immune complex mediated inflammation deposited in the retinal vessels and induce vascular intimal proliferation. This intimal proliferation impacts hemodynamics and destroys laminar flow with blood circulation, predisposing patients to thrombus formation that may cause local ischemia and hypoxia [35]. It has been suggested that, the resistivity index and peak systolic velocity of the ophthalmic artery and central retinal artery present with profound increase in a patient with RA by color Doppler imaging [36]. Therefore, mucinous edema in early retinal vasculopathy may evolve into fibrin-like degeneration, and vascular circulation occlusion due to thrombus formation of small-to-medium-sized vessel [35]. Anyfanti et al found that patients with RA exhibit significantly narrower retinal arterioles than controls, whereas there was no difference in retinal venules [37]. Arterioles occlusion and narrow and diffuse capillary non-perfusion are considered as characteristics of severe vascular occlusion retinopathy [38]. In agreement with our findings and hypothesis, the decrease in the density of S, L, I, SL, SR, IL C1-C5 regions in the macular superficial layer may be a precursor to vascular obliteration retinopathy. The retinal inner layer is supplied by the central retinal arteries, outer four layers is supplied by choroidal vessels, while macula and optic disc is served by choroidal capillaries. According to the result of the non-human primate’s experiment investigating oxygen supply and consumption in the retina, during light adaptation, the retinal circulation of foveal periphery provides an average 11% of the O_2_ supply to retinal photoreceptors and the choroid provides an average 89% of the O_2_ supply [39]. Hence, the outer layer of retina requires higher oxygen and nourishment support which is primarily provided by connective tissue between the sclera and retina. According to Minvielle [40], the deep retinal layer was obviously more affected by these ischemic abnormalities than the superficial retinal layer was. It is believed that the middle retinal layer perfused by the deep retina are a watershed-like region with a high oxygen requirement, making it more prone to ischemia. Choroid, however, could be affected by recurrent attacks of long-term inflammatory disease as a predominant vascular support of the eye, thus affecting vision [41]. Previously reported studies have described that the microvascular prolonged deterioration and subsequent atrophy can lead to choroidal thinning in patients with RA [35,42,43]. Histopathologically, the retinal pigment epithelial lesion due to neutrophilic infiltration, immunocomplex and IgG deposition in the choroid have been confirmed to be the cause of deep retinal layer long-term hypoxia and ischemia in the systemic inflammatory state [44]. This is consistent with our finding that DTMI and DMIR densities in deep retinal layer in RA group were statistically lower than in normal control group. In RA patients, the EDTRS division method indicated statistically decreased microvascular density of the superficial retinal layer in the S, L, and I quadrants, and decreased density of the deep retinal layer only in the S quadrant. For the annular partition method, the microvascular density of deep retinal layer only decreased in the C1, C2, and C4 regions. This phenomenon may be due to the fact that the posterior segment of the eye is nourished by two distinct circulatory systems: central retinal arteries and choroidal vessel. Currently, a various ophthalmology related disease such as diabetes-related macular disease, hypertension-related disease has been demonstrated association with conjunctival microcirculatory abnormality [45,46]. The decreased of conjunctival cupped cells and mucin secretion induced by vascular inflammatory state and immune-mediated stimulation may support out finding of vascular circulatory abnormality observed in RA conjunctiva. The results showed in this study indicated the significantly decreased conjunctival microvascular in RA group and a positive correlation between conjunctival vascular and STMI densities in RA with control groups (correlation coefficient = 0.720, *P*=0.03). We speculate that the branches of the anterior ciliary artery supplying conjunctival nutrition are affected by the deposition of inflammatory mediators, resulting in decreased conjunctival vascular density. Meanwhile, proinflammatory cytokines and chemokines can act on conjunctival microvasculature as angiogenic factors whereas these dysregulated factors may damage the conjunctival and macular retinal microvascular endothelial cells. The immunomodulation of RA patients is dysfunctional, releasing numerous inflammatory cytokines to elevate ESR level. Our finding is that reduction of macular retinal density at superficial and deep layers may be associated with increased ESR, which can be interpreted as a marker of ineffective systemic inflammatory disease control. Inflammatory cytokines, such as interleukin-6 (IL-6) and tumor necrosis factor (TNF) are key regulators under the inflammatory process and have been widely described to be upregulated in patients with RA [47]. A variety of cytokines and chemokines, including IL-6 are constantly produced in synovial fibroblasts after TNF stimulation, and their synergistic interaction with TNF is the pathogenesis of RA [48,49]. The stimulation of TNF and IL-6 can induce the production of numerous F-actin microfilaments as well as reorganization of actin cytoskeleton in vascular endothelial cells (VEC) [50,51]. Enhancing centripetal force and altering cell morphology led to an increase in the endothelial permeability and a disturbance in the distribution of intercellular tight junction proteins, resulting in the dysfunction of the retinal endothelial barrier [52,53]. To the knowledge, retinal VEC assist the inflammatory cascade primarily by expressing adhesion molecules and increasing microvascular permeability. The upregulated TNF increases the expression of vascular cell adhesion protein 1 (VCAM-1 or CD106) in retinal microvessels and augments their binding with endothelial cells, leading to circulatory obstruction and forming a non-perfusion zone [54]. However, in the previous study aimed at elucidating the influence underlying upregulation of the proinflammatory mediators on retinal inflammation, it was found that only the sustained overexpression of TNF initiated an increase in the secretion of additional VCAM-1 as well as proinflammatory cytokines and chemokines [55]. Additionally, continuous overexpressing IL-6 had only minimal effects on the expression level of proinflammatory cytokines in case of ocular involvement [55]. Although the overexpressing TNF, therefore, did cause ocular cellular infiltrates, this was insufficient to result in structural lesions in the front and the back of the eye [55]. While the relationship between retinal vascular density and CRP levels in RA has not been confirmed, elevated CRP level have been indicated to correlate with greater RA disease activity [56]. The evidence from an epidemiological study showed a strong association between an increased CRP level and a high prevalence of cardiovascular comorbidities in patients with RA [57]. A large observational cohort study by Attar et al. [58] has reported that RA patients with a higher level of CRP are more predisposed to atherogenic lipid profile and hyperlipidemia. Additionally, we performed area under the ROC curve analysis of different macular retinal partition methods in the superficial and deep stratifications to detect the implications of RA-induced retinal involvement. The density of IL region of superficial retinal layer indicated the highest positive likelihood ration, while the density of S region had the highest positive likelihood rations in two of layers. This finding highlights the capability of OCTA to distinguish retinal abnormalities caused by RA from those healthy retinas. The retinal microvasculature is the most directly observed blood vessel in RA patients in vivo, and its manifestations can reflect the microcirculation of systemic disease. Alterations in retinal microcirculation in patients with RA may precede discernible joint deformities or ophthalmic diseases. OCTA provides opportunities to early detect these subtle changes, which may be crucial to facilitating early treatment and improving the prognosis of RA patients. In this study, the age- and gender-matched participants with a range of disease severity, and the decreased retinal microvascular densities analyzed in the many regions of superficial and deep layers show that OCTA is a feasible method for distinguishing healthy eyes from those with RA.

Admittedly, there are few limitations in present study should be considered: (1) As a cross-sectional and small sample-sized study, the changes of macular microvascular density along with RA progression and medication usage could not be fully established; (2) We found a worse visual acuity in the RA than in the control group (0.89 ± 0.12 vs. 0.68 ± 0.23, *P*=0.3). Despite the possibility that the inflammation implicating the retina was to blame for the visual impairment of the RA group, the exact pathological mechanism must yet be ascertained; (3) OCTA imaging has its own limitations, namely motion artifacts that can lead to inaccurate interpretation in uncooperative participants. Overall, this cross-sectional study highlights the ability of OCTA to accurately quantify retinal microcirculation alterations before the current detection method, which is referable for the subsequently large sample-sized studies to verify our initial hypothesis. Large-scale and multicentric prospective longitudinal studies are still required to eliminate the limitations and provide a strong basis for transition to clinical practice.

## Conclusion

In the present study, the OCTA results suggested that RA might be associated with retinal microcirculatory changes. Our findings included decreasing the superficial and deep retinal vessel densities, temporal conjunctival capillary density decrease, and a positive correlation between alterations in superficial TMI with conjunctival vessel densities. Additionally, retinal circulatory lesions at the macula could be related to worse visual acuity and even irreversible visual impairment. These findings specified the capability of OCTA to identify the subtle retinal microvessel alterations before the current determination methods. Overall, in those people at high hazards of RA, macular retinal OCTA image is an interesting object that needs to be ascertained, given its potential capacity to early identify retinal involvement.

## Data Availability

The datasets used and/or analyzed during the present study are available from the corresponding author on reasonable request.

## Abbreviations

DMAR: deep macrovascular
DMIR: deep microvascular
DRL: deep retinal layer
ETDRS: Early Treatment of Diabetic Retinopathy Study
FA: fluorescein angiography
HAQ-DI: health assessment questionnaire disability index
OCTA: optical coherence tomography angiography
RA: rheumatoid arthritis
SMAR: **superficial** macrovascular
SMIR: superficial microvascular
TNF: tumor necrosis factor
VCAM: vascular cell adhesion protein 1
VEC: vascular endothelial cells
